# Regulation of Innate Responses during Pre-patent Schistosome Infection Provides an Immune Environment Permissive for Parasite Development

**DOI:** 10.1371/journal.ppat.1003708

**Published:** 2013-10-10

**Authors:** Diana K. Riner, Christine E. Ferragine, Sean K. Maynard, Stephen J. Davies

**Affiliations:** Department of Microbiology and Immunology, Uniformed Services University of the Health Sciences, Bethesda, Maryland, United States of America; Justus-Liebig-University, Germany

## Abstract

Blood flukes of the genus *Schistosoma* infect over 200 million people, causing granulomatous pathology with accompanying morbidity and mortality. As a consequence of extensive host-parasite co-evolution, schistosomes exhibit a complex relationship with their hosts, in which immunological factors are intimately linked with parasite development. Schistosomes fail to develop normally in immunodeficient mice, an outcome specifically dependent on the absence of CD4^+^ T cells. The role of CD4^+^ T cells in parasite development is indirect and mediated by interaction with innate cells, as repeated toll-like receptor 4 stimulation is sufficient to restore parasite development in immunodeficient mice in the absence of CD4^+^ T cells. Here we show that repeated stimulation of innate immunity by an endogenous danger signal can also restore parasite development and that both these stimuli, when administered repeatedly, lead to the regulation of innate responses. Supporting a role for regulation of innate responses in parasite development, we show that regulation of inflammation by neutralizing anti-TNF antibodies also restores parasite development in immunodeficient mice. Finally, we show that administration of IL-4 to immunodeficient mice to regulate inflammation by induction of type 2 responses also restores parasite development. These findings suggest that the type 2 response driven by CD4^+^ T cells during pre-patent infection of immunocompetent hosts is exploited by schistosomes to complete their development to reproductively mature adult parasites.

## Author Summary

Schistosomiasis is a devastating disease caused by *Schistosoma* blood flukes and is a leading parasitic cause of morbidity and mortality in the Developing World. The regulation of inflammatory responses to schistosome eggs trapped in tissues is critical for host survival and is established before egg deposition begins, with the production of the cytokine IL-4 being a hallmark of this process. Here we show that regulation of inflammatory responses also contributes to the development of schistosomes into egg-laying adult parasites. We demonstrate that failure of schistosome development in immunodeficient mice correlates with the absence of the chronic liver inflammation and subsequent immune regulation found in infected wild type mice. Restoration of liver inflammation in immunodeficient mice by repeated administration of liver toxins restored parasite development. Repeated administration of an endogenous inflammatory stimulus also restored parasite development, and also restored aspects of the immune regulation found in wild type mice. Finally, administration of IL-4 alone to immunodeficient animals also restored parasite development and the regulation of inflammation. We propose that schistosomes require immune regulation of inflammation to develop in the hostile immune environment within their hosts. Hence, targeting regulation of inflammation may represent a novel approach to treating or preventing schistosome infections.

## Introduction

As a result of extensive host-parasite co-evolution, helminths exploit resources within their hosts to complete their development and ensure transmission to new hosts. Indeed, most helminths are obligate parasites, requiring the intra-host environment for successful life cycle completion. However, for the most part, the precise host factors that helminths require or utilize, in terms of host cells or molecules, are poorly defined. Previously, CD4^+^ T cells were shown to play a fundamental role in schistosome development [1]–[3], as significant impairment of parasite growth and reproductive activity occurred in mice that lack CD4^+^ T cells. While the precise mechanism by which CD4^+^ T cells mediate this effect is unclear, the mechanism is indirect, as chronic stimulation of innate immune responses with lipopolysaccharide (LPS), a toll-like receptor 4 (TLR4) agonist, during pre-patent infection was able to restore parasite development in the absence of CD4^+^ T cells [4]. Thus, all the host factors necessary for schistosome development are present, or at least can be induced, independently of CD4^+^ T cells. However, whether the mechanisms by which CD4^+^ T cells and chronic LPS stimulation restore schistosome development share any common elements has remained an open question.

Regulation of pro-inflammatory responses is critical for host survival of *S. mansoni* infection [5], and in response to schistosomes and other helminths, the immune system establishes robust T helper 2 (T_H_2) responses that modulate pro-inflammatory processes [6], [7]. In schistosomaisis, T_H_2 responses against parasite antigens are required for the formation of protective granulomas around parasite eggs [8], [9]. T_H_2 responses to worm antigens develop even before the onset of egg production [10], [11] and there is evidence that this immune priming by the developing worms is necessary to ensure proper T_H_2 granuloma formation [12]. T_H_2 responses are also critical for host survival after egg production begins, as lack of IL-4 signaling leads to severe disease and early mortality as a result of excessive pro-inflammatory processes [8], [9], [13]–[15]. Thus, in schistosomiasis, T_H_2 responses serve a dual purpose, to mediate granuloma formation and to regulate inflammation.

Here, we present evidence to suggest that, while fundamentally different, chronic innate responses in immunodeficient mice and adaptive responses in immunocompetent mice ultimately promote parasite development by resulting in a similar outcome, namely the establishment of an immunological milieu where inflammatory processes are regulated. These findings provide insights into the developmental requirements of schistosomes and may identify host dependencies that could be exploited to disrupt schistosome infection.

## Results

### Pre-patent *S. mansoni* infection fails to induce liver inflammation and necrosis in immunodeficient mice

**Figure 1:**
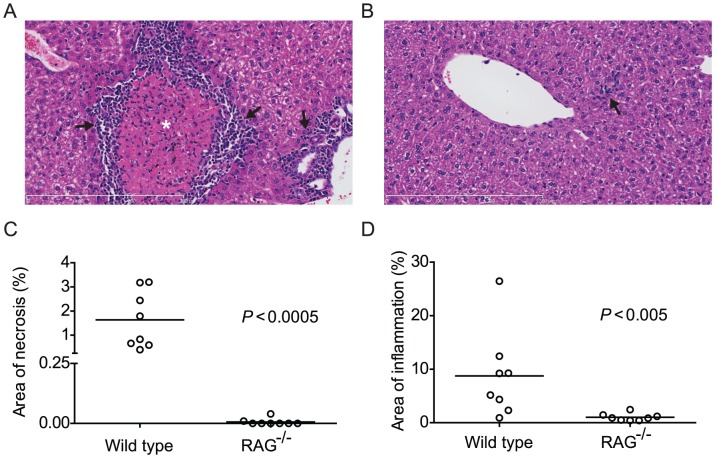
Pre-patent *S. mansoni* infection does not induce liver necrosis and inflammation in RAG^−/−^ mice. Wild type (C57BL/6) and RAG^−/−^ mice were infected with *S. mansoni* cercariae via percutaneous tail exposure and livers were removed for histological analysis at 4 weeks p.i. (A) Representative H&E-stained liver section from a wild type mouse, exhibiting inflammatory cell infiltration (arrows) in periportal area and surrounding a focus of coagulative necrosis (⋆). (B) Representative H&E-stained liver section from a RAG^−/−^ mouse. Note small and isolated cluster of inflammatory cells (arrow). (C) Average percent area of tissue section occupied by coagulative necrosis was calculated for each animal (n = 8, pooled from two independent experiments). Horizontal bars represent mean values for each experimental group. (D) Average percent area of tissue section occupied by inflammatory infiltrate was calculated for each animal (n = 8, pooled from two independent experiments). Horizontal bars represent mean values for each experimental group. *P* values were determined using the Mann Whitney test. Scale bars in A and B are 300 µm in length.

Although chronic LPS stimulation, administered twice weekly during the first six weeks of infection [4], can restore schistosome development in recombination activating gene-deficient (RAG^−/−^) mice, this stimulus is unlikely to be present at high concentrations in mouse plasma during pre-patent infection. We therefore sought to identify other inflammatory processes occurring during the pre-patent stage of schistosome infection in normal mice, as these might be candidates for a physiological stimulus for parasite development. Before the deposition of eggs, worm development in the portal vasculature is associated with both liver inflammation and hepatocellular necrosis of an unknown etiology [16], [17]. To determine whether this pathology also occurs in RAG^−/−^ mice, we compared liver tissue sections from 4 week-infected RAG^−/−^ and wild type mice. As previously reported [16], [17], wild type mice exhibited areas of coagulative necrosis and infiltration with inflammatory cells (Figure 1A). Inflammatory infiltrates, consisting of lymphocytes, eosinophils and mononuclear cells, were located in periportal areas and in the parenchyma, and also surrounded necrotic areas (Figure 1A). In contrast, areas of coagulative necrosis were not seen in the 4 week-infected RAG^−/−^ mice (Figure 1B), with the exception of two animals where small foci of necrosis were detected (data points included in Figure 1C). Furthermore, we observed very little liver inflammation in 4 week-infected RAG^−/−^ mice (Figure 1B), which, when present, was restricted to areas around or near vessel walls (figure 1B). The mean percentage area occupied by necrotic liver tissue was 1.6% for wild type animals, while RAG^−/−^ mice exhibited almost none (Figure 1C). Likewise, the mean percentage area occupied by inflammatory infiltrates was 8.7% in wild type mice, while RAG^−/−^ mice exhibited almost none (Figure 1D). These data show that failure of parasite development in RAG^−/−^ mice correlates with a lack of liver necrosis and inflammation. Furthermore, while the etiology of the liver necrosis in wild type mice is unknown, these data suggest that death of hepatocytes during pre-patent schistosome infection requires an intact adaptive immune system.

### Endogenous danger-associated molecular patterns restore *S. mansoni* development in immunodeficient mice

**Figure 2:**
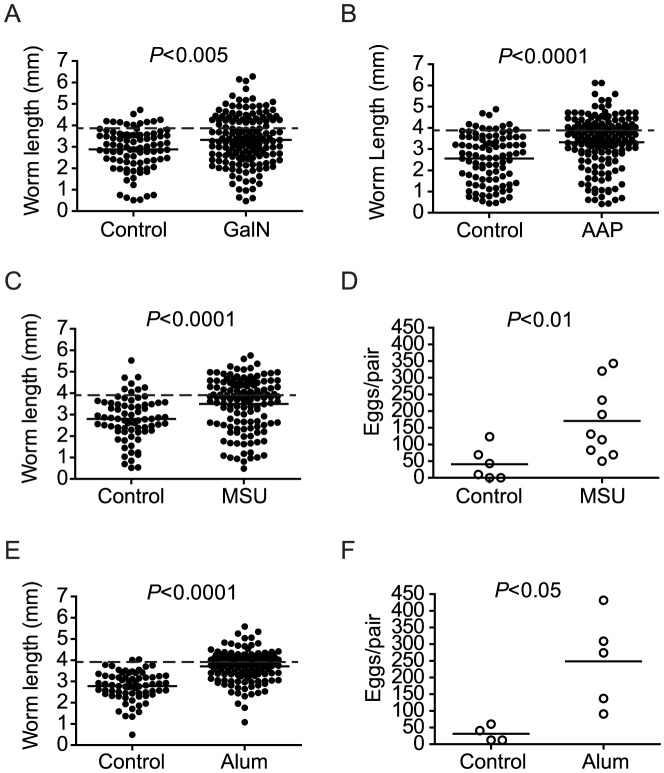
Chronic hepatocyte injury or DAMP administration facilitates *S. mansoni* development in RAG^−/−^ mice. Groups of RAG^−/−^ mice were infected with *S. mansoni* and treated throughout pre-patent infection with the indicated hepatotoxins or inflammatory stimuli, as described in Materials and Methods. Parasites were perfused from the portal tract and livers removed for egg enumeration at 6 weeks p.i. (A) Length of male worms recovered from RAG^−/−^ mice chronically treated with D-(+)-galactosamine hydrochloride (GalN). (B) Length of male worms recovered from RAG^−/−^ mice chronically treated with acetaminophen (AAP). (C and E) Length of male worms and (D and F) liver egg burdens from RAG^−/−^ mice chronically treated with monosodium urate (MSU; C and D) or alum (E and F). Horizontal bars represent mean values for each experimental group. Groups of 4 to 5 mice were used for each experimental condition. *P* values for worm lengths determined using student's T-test with Welch's correction. *P* values for egg production determined using the Mann Whitney test. Data shown for the MSU experiment are pooled from two independent experiments. Dashed lines in A, B, C, and E indicate the average length of male *S. mansoni* worms recovered from wild type mice at day 42 post infection (3.9 mm).

In view of the fact that a failure of parasite development in RAG^−/−^ mice correlates with a lack of liver necrosis and inflammation, we hypothesized that induction of liver necrosis and inflammation would restore parasite development in these animals. To test this hypothesis, we administered two well-characterized hepatotoxins, acetaminophen (AAP) or D-galactosamine (GalN), to RAG^−/−^ mice throughout pre-patent infection, at doses sufficient to result in hepatocellular death and inflammation [18]–[21], in an attempt to simulate the cell death observed in wild type mice. While chronic hepatotoxin treatment did not recapitulate the coagulative necrosis seen in wild type mice, both treatments induced histological evidence of widespread hepatocellular damage and inflammation (supplementary Figure S1). Furthermore, by six weeks post infection (p.i.), both treatments partially restored parasite growth when compared to control RAG^−/−^ animals that received vehicle alone (Figure 2A and 2B), while parasite egg production was unaffected (essentially none, data not shown). These results suggested that liver necrosis and inflammation played a role in modulating schistosome development.

Cellular injury results in the release of uric acid into the extracellular environment that crystallizes to form monosodium urate (MSU) [22], an endogenous danger-associated molecular pattern (DAMP) that activates the NALP3 inflammasome [23]. Since chronic hepatotoxin treatment partially restored parasite development in RAG^−/−^ mice, we hypothesized that DAMP-mediated inflammatory processes would also restore parasite development in these animals. To test this hypothesis, we administered MSU to infected RAG^−/−^ mice throughout the first six weeks of infection and compared worm development to that in control RAG^−/−^ mice that received vehicle alone. Treatment with MSU resulted in robust restoration of parasite growth (Figure 2C) and partial restoration of egg production (Figure 2D) in RAG^−/−^ mice suggesting that, like LPS, chronic DAMP-mediated inflammation can also stimulate parasite development. In further support of a role for inflammasome-mediated inflammation in stimulating parasite development, we also found that treatment with alum, an exogenous NALP3 inflammasome agonist [24], [25], also resulted in robust restoration of parasite growth (Figure 2E) and partial restoration of egg production (Figure 2F) in RAG^−/−^ mice. Taken together, these data suggest that, like the exogenous danger signal LPS, endogenous danger signals that stimulate inflammation via inflammasomes can also stimulate schistosome development.

### Parasite development correlates with regulation of IL-1β transcription

**Figure 3:**
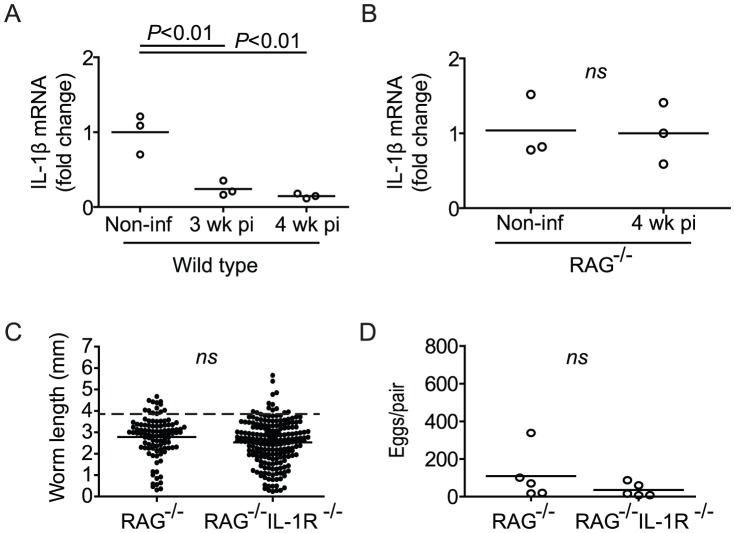
Impaired *S. mansoni* development in RAG^−/−^ mice correlates with maintenance of IL-1β transcription. (A and B) Groups of wild type (A) and RAG^−/−^ mice (B) were infected percutaneously with *S. mansoni* cercariae, euthanized at the indicated time points and spleens removed for analysis of IL-1β mRNA levels by real-time PCR. Horizontal bars represent mean values for each experimental group. For wild type mice (A), *P* values were calculated by ANOVA with Tukey's post-test. For RAG^−/−^ mice (B), *P* value was calculated using the Mann Whitney test. (C) Length of male worms and (D) liver egg burdens from *S. mansoni*-infected RAG^−/−^ and RAG^−/−^ IL-1R^−/−^ mice at 6 weeks p.i. Horizontal bars represent mean values for each experimental group. *P* values were determined using student's T-test with Welch's correction and the Mann Whitney test. Dashed line in C indicates the average length of male *S. mansoni* worms recovered from wild type mice at day 42 post infection (3.9 mm).

By serving as a molecular platform for caspase 1 activation, inflammasomes drive IL-1β-mediated inflammation by catalyzing the conversion of inactive pro-IL-1β to the bioactive form [26]. As two different inflammasome agonists restored parasite development in RAG^−/−^ mice, we hypothesized that IL-1β-mediated inflammation may be implicated in parasite development. To address this issue, we first examined IL-1β mRNA levels during pre-patent infection of wild type mice. Unexpectedly, we found that steady-state splenic mRNA levels of IL-1β in wild type mice at 3 and 4 weeks p.i. were down-regulated compared to the baseline levels found in non-infected control mice (Figure 3A). In contrast, IL-1β mRNA levels remained unchanged in the spleens of 4 week-infected RAG^−/−^ mice when compared to non-infected controls (Figure 3B). Thus, normal parasite development correlated with down-regulation of steady-state IL-1β transcription. To test whether a failure to down-regulate IL-1β signaling in RAG^−/−^ mice is the cause of impaired schistosome development in these animals, we infected RAG^−/−^ IL-1R^−/−^ knockout mice, predicting that, if this were the case, ablation of IL-1 signaling would restore parasite development in a RAG-deficient context. However, worms recovered from RAG^−/−^ IL-1R^−/−^ mice 6 weeks p.i. did not differ significantly from those obtained from RAG^−/−^ mice, being small in size (Figure 3C) and reproductively inactive (Figure 3D). Therefore, parasite development correlates with regulation of IL-1β transcription, but IL-1R signaling is not directly responsible for inhibiting parasite development in RAG^−/−^ mice.

### Chronic innate immune stimulation with LPS or MSU results in down-regulation of pro-inflammatory signals

**Figure 4:**
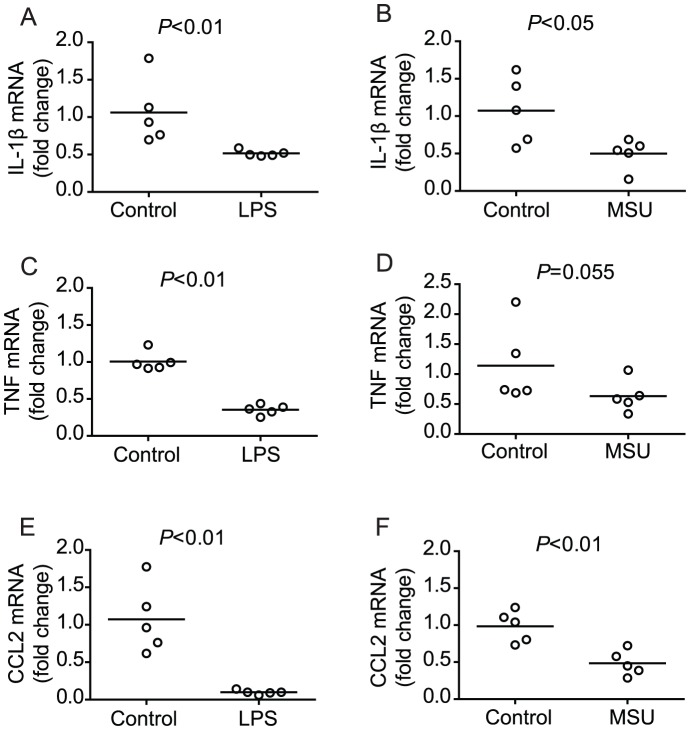
Chronic LPS or MSU administration restores regulation of pro-inflammatory cytokine transcription in infected RAG^−/−^ mice. Groups of *S. mansoni*-infected RAG^−/−^ mice were repeatedly treated throughout pre-patent infection with either LPS (A, C, E) or MSU (B, D, F), as described in Materials and Methods. At 6 weeks p.i., mice were euthanized and spleens removed for analysis of IL-1β (A and B), TNF (C and D) and CCL2 (E and F) mRNA content by real-time PCR. Horizontal bars represent mean values for each experimental group. *P* values were calculated using the Mann Whitney test.

As our examination of IL-1β transcription in wild type and RAG^−/−^ mice revealed that normal schistosome development correlated with down-regulation of IL-1β transcription in wild type mice, we next examined the effect of the LPS and MSU treatment regimens on IL-1β transcription in RAG^−/−^ mice, as both treatments restore parasite development in these animals. Repeated treatment with LPS (Figure 4A) or MSU (Figure 4B) throughout the first six weeks of infection resulted in down-regulation of splenic IL-1β mRNA levels in infected RAG^−/−^ mice by week six p.i., similar to the down-regulation seen in infected wild type mice (Figure 3A). Thus, while down-regulation of IL-1β transcription is mediated by the adaptive immune system in wild type mice, chronic administration of LPS or MSU to RAG^−/−^ mice can also result in IL-1β down-regulation, in the absence of an adaptive immune system. Furthermore, chronic LPS and MSU treatments resulted in down-regulation of other pro-inflammatory signals, as evidenced by reduced splenic mRNA levels for TNF (Figure 4C and 4D) and CCL2 (Figure 4E and 4F), a chemokine important for inflammatory macrophage recruitment [27]–[29]. Finally, we found that transcriptional down-regulation of pro-inflammatory genes to levels lower than those in control animals required chronic exposure to MSU (supplementary Figure S2 A–C), as transcription of pro-inflammatory genes peaked rapidly following a single injection of MSU (data not shown) and then returned to the levels observed in non-treated mice by 18 hours post injection (supplemental Figure S2 D–F).

### Parasites fail to develop when pro-inflammatory gene transcription is sustained

**Figure 5:**
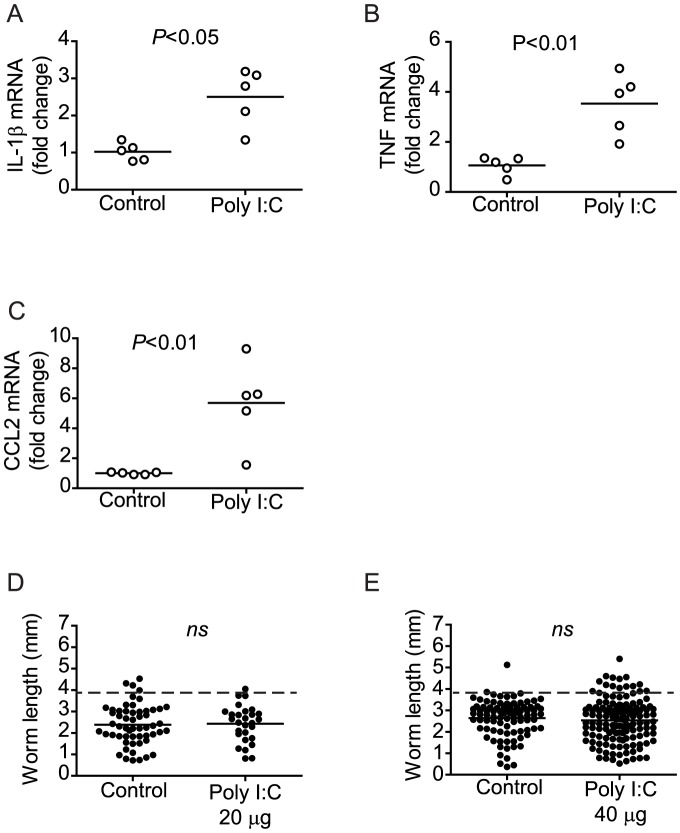
Chronic poly I:C administration does not restore regulation of pro-inflammatory cytokine transcription or parasite development in infected RAG^−/−^ mice. Groups of *S. mansoni*-infected RAG^−/−^ mice were repeatedly treated throughout pre-patent infection with poly I:C, as described in Materials and Methods. At 6 weeks p.i., mice were euthanized, parasites perfused from the portal tract and spleens removed for analysis of IL-1β (A), TNF (B) and CCL2 (C) mRNA content by real-time PCR. (D and E) Length of male worms recovered from RAG^−/−^ mice treated repeatedly with 20 µg (D) or 40 µg (E) doses of poly I:C. Horizontal bars represent mean values for each experimental group. *P* values were calculated using the Mann Whitney test and student's T-test with Welch's correction. Dashed lines in D and E indicate the average length of male *S. mansoni* worms recovered from wild type mice at day 42 post infection (3.9 mm).

Our analysis of wild type, RAG^−/−^ and MSU- and LPS-treated RAG^−/−^ mice showed that normal parasite development correlates with the overall down-regulation of pro-inflammatory gene transcription. However, we considered the possibility that elevated pro-inflammatory gene transcription early in the course of LPS or MSU treatment, before regulation was induced, could be the factor important for stimulating parasite development, rather than the ultimate down-regulation of these genes. To explore this possibility, we sought to identify comparable treatments where chronic administration of an inflammatory stimulus did not result in down-regulated pro-inflammatory gene transcription. To this end, we found that administration of poly I:C [30], a TLR3 ligand, to RAG^−/−^ mice throughout the first six weeks of infection, resulted in up-regulation of splenic mRNA for IL-1β, TNF, and CCL2 (Figure 5 A–C), rather than their down-regulation. Consistent with a role for pro-inflammatory gene down-regulation in permitting schistosome development, chronic administration of poly I:C also failed to restore parasite development in RAG^−/−^ mice, as worms recovered from treated animals did not differ significantly in size (figure 5D–E) or egg output (essentially none, data not shown) from vehicle-treated controls at either of the two doses tested.

### TNF blockade restores parasite development in immunodeficient mice

**Figure 6:**
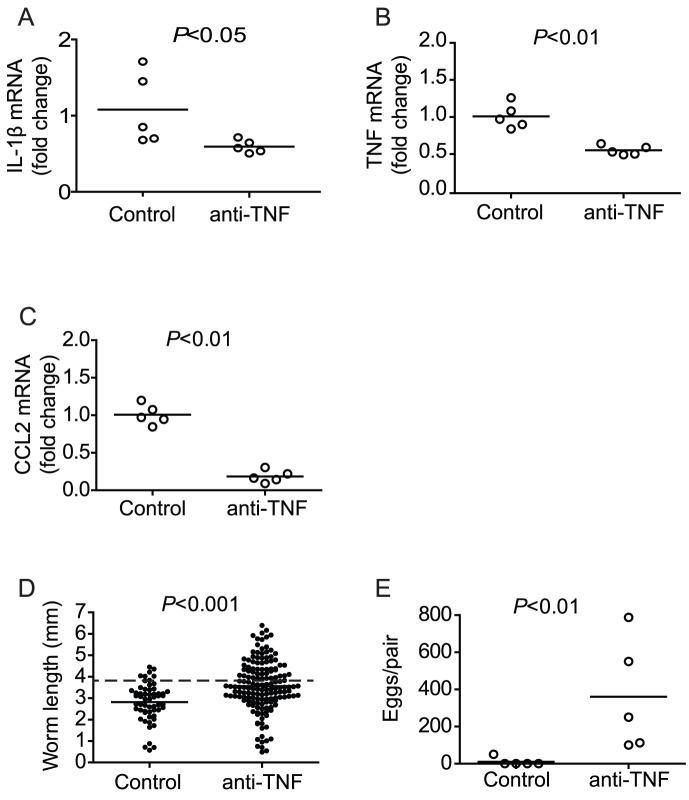
Chronic administration of anti-TNF antibody restores regulation of pro-inflammatory cytokine transcription and parasite development in infected RAG^−/−^ mice. Groups of *S. mansoni*-infected RAG^−/−^ mice were repeatedly treated throughout pre-patent infection with anti-TNF antibody, as described in Materials and Methods. At 6 weeks p.i., mice were euthanized, parasites perfused from the portal tract and spleens removed for analysis of IL-1β (A), TNF (B) and CCL2 (C) mRNA content by real-time PCR. (D) Length of male worms and (E) liver egg burdens from RAG^−/−^ mice treated with anti-TNF antibody. Horizontal bars represent mean values for each experimental group. *P* values were calculated using the Mann Whitney test and student's T-test with Welch's correction. Dashed line in D indicates the average length of male *S. mansoni* worms recovered from wild type mice at day 42 post infection (3.9 mm).

As restoration of parasite development in RAG^−/−^ mice correlated with down-regulation of pro-inflammatory gene transcription (in LPS- and MSU-treated RAG^−/−^ mice) and was unaltered when pro-inflammatory gene transcription was sustained (in poly I:C-treated RAG^−/−^ mice), we hypothesized that it was the down-regulation of pro-inflammatory genes that permits parasite development to proceed. To test this hypothesis, we attempted to suppress pro-inflammatory gene activity in RAG^−/−^ mice by blocking TNF signaling with a neutralizing antibody, which has been shown to decrease IL-1β production in models of sepsis [31] and arthritis [32]. Administration of the anti-TNF antibody did not cause acute increases in pro-inflammatory gene transcription (data not shown), and its administration throughout pre-patent infection led to the down-regulation of IL-1β, TNF, and CCL2 transcription in the spleens of RAG^−/−^ mice by four weeks p.i. (Figure 6A–C), similar to that observed in wild type mice and with chronic LPS or MSU treatment in RAG^−/−^ mice. Furthermore, anti-TNF treatment resulted in significant increases in parasite size (Figure 6D) and reproductive activity (Figure 6E) when compared to control RAG^−/−^ mice. Thus, these data supported our hypothesis that schistosome development requires the down-regulation of pro-inflammatory gene transcription during pre-patent infection.

### Administration of IL-4 complex restores parasite development in immunodeficient mice

**Figure 7:**
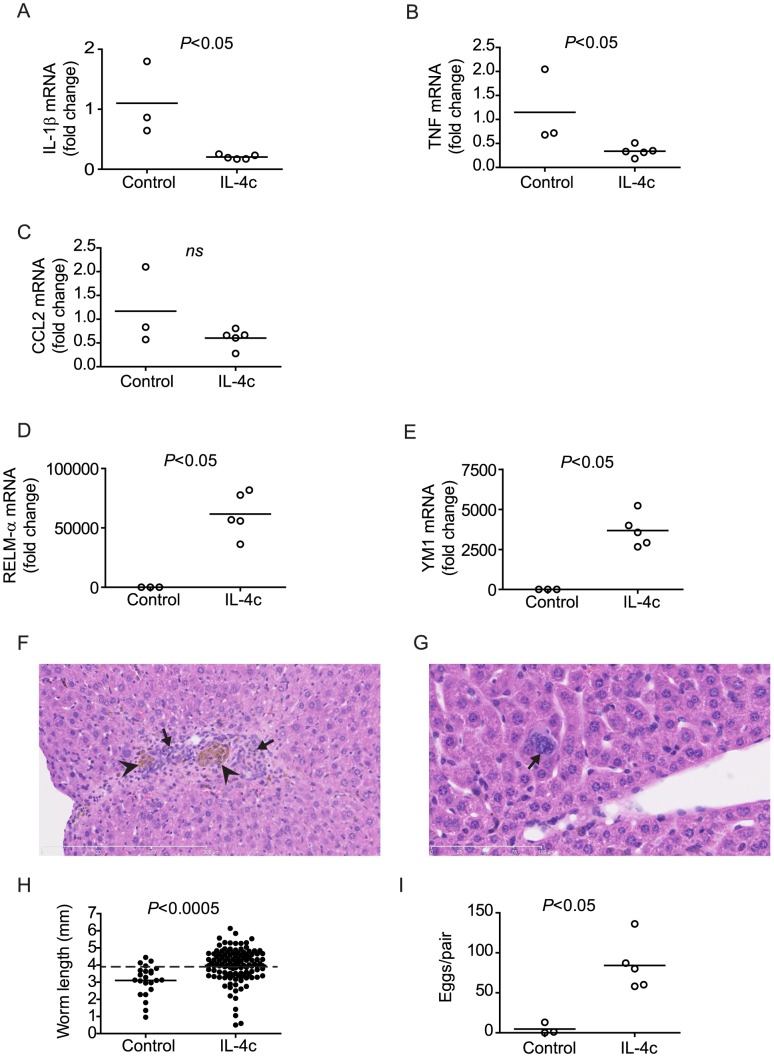
Chronic administration of IL-4 restores regulation of pro-inflammatory cytokine transcription and parasite development in infected RAG^−/−^ mice. Groups of *S. mansoni*-infected RAG^−/−^ mice were repeatedly treated throughout pre-patent infection with IL-4 complex (IL-4c), as described in Materials and Methods. At 6 weeks p.i., mice were euthanized, parasites perfused from the portal tract and spleens (A–C) and livers (D, E) removed for analysis of IL-1β (A), TNF (B), CCL2 (C), RELM-α (D) and YM1 (E) mRNA content by real-time PCR. (F and G) Representative low- (F; 20×) and high-power (G; 40×) fields of H&E-stained liver sections from infected RAG^−/−^ mice treated with IL-4c. In (F), note inflammatory infiltrate (arrows), composed partly of mononuclear phagocytes containing brown by-product of schistosome hemoglobin degradation (arrowheads). In (G), arrow indicates a multinucleate giant cell. Lengths of scale bars are 300 µm (F) and 100 µm (G). (H) Length of male worms and (I) liver egg burdens from RAG^−/−^ mice treated with IL-4. Horizontal bars represent mean values for each experimental group. *P* values were calculated using the Mann Whitney test and student's T-test with Welch's correction. Dashed line in H indicates the average length of male *S. mansoni* worms recovered from wild type mice at day 42 post infection (3.9 mm).

In *S. mansoni-*infected wild type mice, down-regulation of IL-1β transcription occurs via an adaptive immune mechanism, as there is a failure of IL-1β mRNA down-regulation when the adaptive immune system is ablated (Figure 3). In previous studies, we showed that, prior to the onset of egg production, pre-patent schistosome infection results in the rapid establishment of a T_H_2 response [10], [11], where CD4^+^ T cells produce significant quantities of IL-4 in response to worm antigens [10]. As IL-4 is a type 2 cytokine that regulates pro-inflammatory signals, including IL-1β [33]–[35], we hypothesized that IL-4 may represent the adaptive mechanism by which IL-1β is regulated in wild type mice. To test whether IL-4 was sufficient to regulate pro-inflammatory gene transcription in RAG^−/−^ mice, we administered IL-4 complex (IL-4c) to infected RAG^−/−^ mice during pre-patent infection and examined pro-inflammatory gene transcription in the spleen at week six p.i. Administration of IL-4c resulted in down-regulation of IL-1β and TNF transcription in the spleens of treated RAG^−/−^ mice (Figure 7A and 7B), similar to that observed in wild type mice and RAG^−/−^ mice treated with LPS, MSU or anti-TNF. Transcription of CCL2 was also reduced by IL-4c treatment, although the difference between treated and control animals was not significant (Figure 7C). The transcription of RELM-α and YM1, both markers of alternative macrophage activation [36], was dramatically up-regulated in the spleens (data not shown) and the livers of IL-4c-treated animals (Figure 7D and 7E), suggesting that IL-4c treatment induced an innate type 2 response and the accumulation of alternatively activated (M2) macrophages in livers of RAG^−/−^ mice. Indeed, administration of IL-4c also restored the accumulation of mononuclear cells in the livers (Figure 7F) of infected RAG^−/−^ mice and induced giant cell formation (Figure 7G), a previously reported hallmark of alternatively activated macrophage responses [37]. Finally, administration of IL-4c resulted in the restoration of parasite growth (Figure 7H) and reproductive activity (Figure 7I). Our data demonstrate that IL-4, a type 2 cytokine produced as part of the adaptive immune response to pre-patent schistosome infection, is sufficient to regulate pro-inflammatory signals and restore schistosome development and may represent the mechanism by which CD4^+^ T cells permit normal parasite development in wild type mice.

### Regulation of pro-inflammatory signals, not M2 responses, restores parasite development

**Figure 8:**
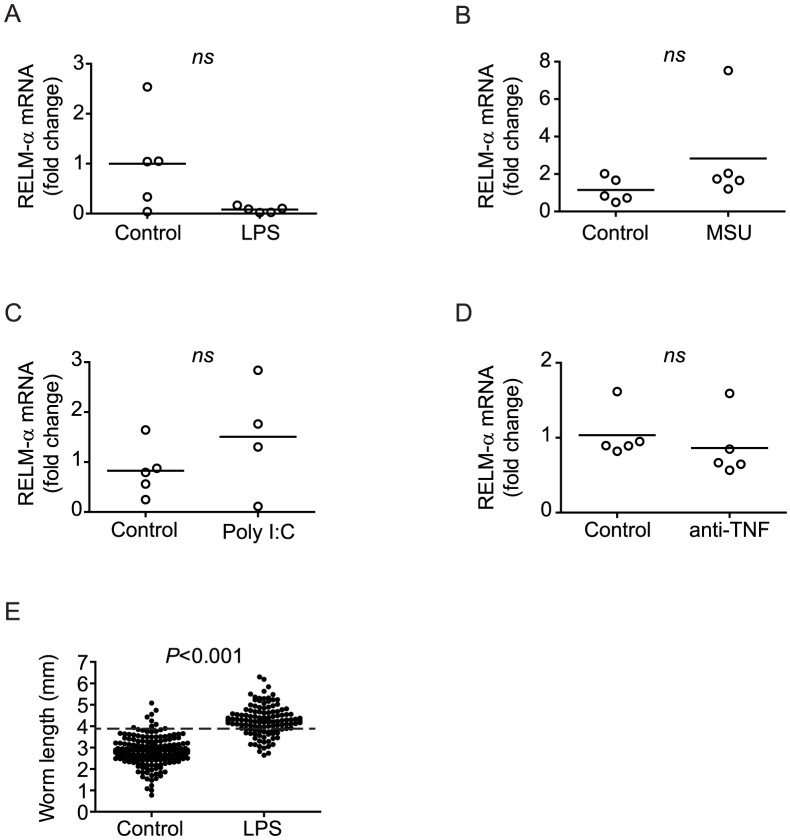
Restoration of parasite development is not dependent on M2 responses. Groups of *S. mansoni*-infected RAG^−/−^ mice were repeatedly treated throughout pre-patent infection with either LPS (A) or MSU (B), poly I:C (C) or anti-TNF antibody (D) as described in Materials and Methods. At 6 weeks p.i., mice were euthanized and livers removed for analysis of RELM-α mRNA content by real-time PCR. E, groups of RAG^−/−^/γ_c_^−/−^ were infected with *S. mansoni* and repeatedly treated throughout per-patent infection with LPS or PBS (control), as described in Materials and Methods. Parasites were perfused from the portal system at 6 weeks p.i. and the length of the male worms measured. Horizontal bars represent mean values for each experimental group. *P* values were calculated using the Mann Whitney test. Dashed line in E indicates the average length of male *S. mansoni* worms recovered from wild type mice at day 42 post infection (3.9 mm).

Our finding that IL-4 administration restored parasite development in RAG^−/−^ mice, in a manner similar to chronic LPS or MSU administration and TNF blockade, prompted us to examine whether these other treatments, like IL-4, promoted M2 conditioning of macrophages, perhaps by stimulating release of endogenous IL-4 from innate immune cells. However, levels of RELM-α transcript in the liver were unchanged by either chronic PAMP (LPS, poly I:C) or DAMP (MSU) treatment, or by TNF blockade (Figure 8A–D), suggesting these treatments do not induce M2 responses and that parasite development does not require M2 conditioning of macrophages per se. To further test the potential involvement of innate sources of IL-4 in restoring parasite development during chronic immune stimulation, we tested whether LPS could restore schistosome development in RAG^−/−^ mice that are also deficient for the common γ chain (γ_c_) [38], a critical component of the IL-4 receptor complex. Chronic LPS treatment of infected RAG^−/−^/γ_c_^−/−^ mice restored parasite growth to levels comparable with those observed in LPS-treated RAG^−/−^ mice (Figure 8E), indicating that IL-4 signaling is not required for restoration of schistosome development by chronic LPS administration. Together, these findings suggest it is the regulation of pro-inflammatory processes by IL-4, rather than IL-4-driven M2 macrophage responses, that is critical in permitting schistosome development to proceed.

## Discussion

Numerous lines of evidence indicate that type 2 responses are beneficial in schistosome infection, not because these responses mediate immunity against schistosomes but because they limit potentially damaging pro-inflammatory responses. For example, in mice deficient in IL-4, IL-4 and IL-10, IL-4 and IL-13 or IL-4 receptor, decreased host survival is observed during acute schistosome infection due to excessive pro-inflammatory cytokine expression and increased liver and intestinal pathology [8], [9], [14], [39]. Likewise, in schistosomiasis patients, severe disease is correlated with decreased production of type 2 cytokines and elevated levels of IFN-γ, TNF and nitric oxide [40]. However, there is also evidence that type 2 responses ultimately benefit schistosomes, and that this benefit extends beyond the obvious relationship between extended host survival and the increased likelihood of transmission to snail intermediate hosts. For example, it has long been recognized that egress of schistosome eggs across the bowel wall is immune-dependent [41]. Subsequent macrophage-specific ablation of IL-4R expression showed that IL-4/IL-13-responsive macrophages are specifically required for egg passage into the intestinal lumen [39]. These observations suggest that host-parasite co-evolution has not only selected for immune responses that prolong host survival, but also for parasites that are able to take advantage of the resulting immunological milieu. The data we present here support the hypothesis that control of pro-inflammatory signals may also be intimately linked to parasite development before the onset of egg production.

Our finding that chronic stimulation with LPS could restore schistosome development in RAG^−/−^ mice presented a paradox, as there is no obvious parallel between the inflammatory response to LPS and the response induced by pre-patent schistosome infection in wild type mice. However, inflammation, albeit in response to necrotic hepatocytes, is a feature of pre-patent schistosome infection in immune-competent, but not RAG^−/−^ mice, suggesting there is a link between inflammation and normal parasite development. In support of this hypothesis, we show that restoration of DAMP-mediated inflammation in RAG^−/−^ mice also restored parasite development. While endogenous DAMPs stimulate inflammation by pathways distinct from exogenous PAMPs such as LPS [42], our finding that both can restore parasite development suggests it is the inflammation itself rather than the inciting cause that is relevant to parasite development. The contribution of necrosis-induced inflammation to promoting parasite development in wild type mice is an interesting and unresolved question. One way to address this question may be to inhibit necrosis in wild type mice and examine for effects on parasite development. Identification of the mechanism leading to hepatocellular necrosis in wild type mice may make this approach possible. The absence of necrosis in RAG^−/−^ mice suggests that adaptive responses are involved in necrosis induction. Alternatively, the lack of necrosis in these animals may be a result of diminished parasite growth, rather than a cause. However, we have not observed hepatocellular necrosis in RAG^−/−^ mice where parasite development is restored by LPS treatment (data not shown), suggesting that developing parasites do not directly cause liver necrosis.

Our observation that steady state transcription of the pro-inflammatory cytokine IL-1β is down-regulated in infected wild type mice, but not in RAG^−/−^ animals, further suggested a role for inflammatory processes in schistosome development, but in an inhibitory capacity. However, the non-permissiveness of RAG^−/−^ mice for parasite development is not specifically due to a failure to down-regulate IL-1 signaling, as ablation of IL-1R activity in a RAG^−/−^ context did not restore parasite development. This result led us to hypothesize that parasite development may require a more global regulation of pro-inflammatory processes and that decreased IL-1β transcription was simply a correlate of this regulation. If this was correct, we predicted that restoration of parasite development in RAG^−/−^ mice by chronic LPS and MSU administration would be associated with transcriptional regulation of IL-1β and other pro-inflammatory genes. The induction of LPS tolerance in response to repeated LPS exposure is a well-recognized negative feedback mechanism thought to be mediated by a variety of mechanisms, including regulation of downstream protein kinases, resulting in down-regulation of inflammatory cytokine transcription [43]. We show here that chronic MSU exposure also results in down-regulation of pro-inflammatory gene transcription. While MSU signals via pathways distinct from LPS [44], the existence of negative feedback mechanisms that regulate persistent MSU signaling is not unexpected and evidence for the induction of regulatory mechanisms by endogenous DAMPs exists. For example, toxicological injury to the liver by AAP first results in an early pro-inflammatory response dominated by classically activated (M1) macrophages, but this initial response is followed by suppression of the initial pro-inflammatory response and promotion of wound healing by immunoregulatory, alternatively activated (M2) macrophages [45], [46]. Second, both alum and uric acid have been shown to promote T_H_2 immunity and suppression of pro-inflammatory processes through NALP3 independent mechanisms [47], [48]. Similarly, LPS tolerance is associated with induction of immunoregulatory M2 macrophages [49]. Based on our findings, we suggest that LPS and MSU restore parasite development in RAG^−/−^ mice by virtue of their ability to induce regulation of pro-inflammatory signals when administered chronically.

To further test whether inflammation per se, or the regulation that results from the inflammation was required for parasite development, we sought to identify inflammatory stimuli that did not lead to regulation. We found that the TLR3 ligand poly I:C, even when administered repeatedly under the same regimen as LPS or MSU, failed to reduce baseline levels of inflammatory gene transcription, resulting instead in overall elevated levels of transcription, even 18 hours post administration of the final dose. Unlike other TLR ligands like LPS, poly I:C does not stimulate MyD88-dependent signaling, utilizing instead a TRIF-dependent pathway that appears not to be subject to the same negative feedback regulation [50]. Chronic poly I:C administration therefore afforded us the opportunity to examine schistosome development in the context of persistent inflammation without the associated regulation. Consistent with a role for regulation in parasite development rather than inflammation, chronic administration of poly I:C, at two different doses, failed to enhance parasite development.

Because poly I:C signaling is mediated via a distinct receptor and adapter molecule [50], we cannot exclude the possibility that some essential component of the response induced by MSU or LPS is absent from the response to poly I:C. However, we reasoned that if regulation of inflammation was the critical element in permitting parasite development, then direct regulation of inflammation in the absence of exogenous inflammatory stimuli would also be able to restore schistosome development. The success of anti-TNF neutralization therapy in controlling inflammatory disorders is due to the ability of this intervention to broadly control inflammation, mediated by TNF and associated signals, including IL-1β [32]. In the absence of any additional inflammatory stimuli, administration of anti-TNF antibodies to infected RAG^−/−^ mice recapitulated the regulation of inflammatory gene transcription observed after chronic LPS or MSU administration and also restored schistosome development, lending further support to our conclusion that regulation of inflammation is required for normal parasite development.

In an immunocompetent host, pre-patent schistosome infection induces a T_H_2 response, characterized by production of IL-4 by CD4^+^ T cells in response to schistosome antigens [10]. In addition to driving T_H_2 effector mechanisms such as antibody isotype class switch recombination in B cells and M2 macrophage development, IL-4 also regulates pro-inflammatory signals [33] and is therefore a likely regulator of pro-inflammatory processes during pre-patent schistosome infection. Consistent with this role, IL-4 administration to RAG^−/−^ mice was sufficient to restore regulation of IL-1β and TNF transcription and induce type 2 responses, as evidenced by the dramatic up-regulation of RELM-α and YM1 transcripts. Significantly, IL-4 treatment also resulted in increased schistosome growth and egg production, demonstrating that a single type 2 cytokine was sufficient to significantly augment parasite development. From these results, it is tempting to speculate that regulation mediated by IL-4 may be the principle contribution of the adaptive response in wild type mice to permitting parasite development to proceed. However, IL-4 is unlikely to be the only adaptive immune factor to promote schistosome development, as parasite development proceeds normally when IL-4 signaling is blocked in otherwise immunologically intact mice, whether by anti-IL-4 antibody or through genetic disruption [1]. Furthermore, we previously showed that CD4^+^ T cells that lack specificity for schistosome antigens and cannot respond to schistosome infection can still positively influence parasite development [4]. There is no evidence this non-cognate effect requires IL-4, but is likely a consequence of homeostatic interactions between CD4^+^ T cells and innate antigen-presenting cells that modulate myeloid cell function [4]. Thus, there is likely considerable latitude in the requirement of schistosomes for regulation, as illustrated by the diversity of mechanisms by which parasite development can be restored in RAG^−/−^ mice (chronic administration of LPS or MSU, or administration to anti-TNF antibody or recombinant IL-4). However, it remains a possibility that the host elements required for schistosome development may be common to the responses induced by each of these mediators.

As there is evidence that alternatively activated M2 macrophage rather than classically activated M1 macrophage responses are favored under conditions of chronic immune stimulation [49], [51]–[55], it is tempting to hypothesize that M2 macrophages are critical for parasite development. However, we could find no evidence that our repeated treatment of RAG^−/−^ mice with LPS, MSU or anti-TNF antibody led to M2 induction. Thus, while the type 2 response and possibly M2 macrophages may represent host factors that schistosomes co-opt to complete development in immunologically intact mice, our data suggest there are other more fundamental aspects of innate responses that schistosomes exploit, rather than M2 macrophages per se. For example, LPS tolerization and alternative macrophage activation induce overlapping changes in macrophage function, including the down-regulation of pro-inflammatory mediator expression and production of toxic reactive oxygen intermediates, that in both scenarios is mediated by a common regulatory pathway that involves NF-κB p50 [49]. Thus, it is possible that schistosomes have evolved to take advantage of the regulatory aspects of type 2 responses that modulate pro-inflammatory responses.

Why regulation of pro-inflammatory signals would influence schistosome development remains to be determined. Allen and Wynn recently suggested that T_H_2 immunity evolved in order to supply a rapid response that repairs the tissue damage generated by helminths [56], highlighting that the need for regulation of innate immune responses is not limited to schistosome infections, but is a common feature of immune responses directed towards tissue-penetrating helminths. For example, M2 macrophages and T_H_2 responses are critical for limiting lung tissue damage after experimental *Nippostrongylus brasiliensis* infection [57], by controlling initial IL-17-driven inflammatory responses and promoting resolution of tissue damage [58]. M2 macrophages have also been shown to limit brain tissue pathology in a murine model of neurocysticercosis [59] [60], where decreased numbers of brain M2 macrophages in *Mesocestoides corti*-infected mice was shown to result in increased disease severity. The induction of systemic T_H_2 responses has been shown to occur early in *S. mansoni* infection [10], [11]. Indeed, multiple exposure of skin to invading cercariae, as likely occurs under field conditions, has been shown to be sufficient to induce M2 conditioning of macrophages at the site of infection [61]. Thus, type 2 responses are induced sufficiently early during infection to exert an effect on the developing schistosomes. There is already evidence that schistosomes require M2 macrophages later in infection, as these cells are critical for the egress of schistosome eggs from the body of the host [39], and are required for host survival after egg production begins [8], [13]–[15]. M2 macrophages in particular are of critical importance in the regulation of excessive egg-induced inflammation and the lack of M2 macrophages during acute infection is lethal, as shown by the macrophage-specific ablation of IL-4Rα expression [39]. As M2 macrophages are a specific hallmark of the host response to schistosomes and other helminths [56], the hypothesis that schistosomes have evolved to specifically exploit this aspect of the host response is an attractive one.

In addition to regulating inflammatory processes that may damage developing schistosomes, there are other mechanisms by which type 2 or regulatory responses might contribute to schistosome development. As mediators of tissue repair and remodeling, one possibility is that M2 or immunomodulatory macrophages mediate critical niche remodeling in portal venules where the rapidly growing schistosomes reside, akin to the lymphatic vascular remodeling induced by filarial nematodes [62]. Alternatively, the presence of M2 or immunomodulatory macrophages may alter the availability of host-derived nutrients or other molecules that the developing parasites require [63]. Macrophage activation status is associated with profound changes in cell metabolism [64] that could influence the concentrations of host factors in the immediate vicinity of larval schistosomes. Finally, molecules associated with immunoregulation and type 2 responses may be directly recognized by schistosomes and utilized as signals of an environment that is appropriate for parasite development. A somewhat similar relationship was recently proposed to influence the development of *Litomosoides sigmodontis*, a filarial nematode, which accelerates its larval development and produces greater numbers of microfilaria in response to IL-5 and eosinophils [65]. It was suggested that these parasites utilize IL-5 as a predictor for future survival and altered life expectancy [65]. Studies to examine these and other possibilities in the context of schistosome infection are currently underway.

Here we presented evidence that regulation of pro-inflammatory processes is a contributor to determining the developmental fate of schistosomes in their definitive mammalian host. It remains to be determined how schistosomes might recognize a regulated immune environment and how this environment influences parasite development. However, these findings suggest that inflammation and its regulation are key components of a host environment permissive to schistosome infection. Thus, modulation of inflammatory processes may be a useful approach to disrupting schistosome infection and could lead to new insights for improved treatments or vaccines for schistosomiasis.

## Materials and Methods

### Ethics statement

All animal studies were performed in strict accordance with the recommendations of the Office of Laboratory Animal Welfare at the National Institutes of Health and the USUHS Institutional Animal Care and Use Committee. All animal protocols were reviewed and approved by the USUHS Institutional Animal Care and Use Committee, permit number A3448-01.

### Experimental animals

RAG-1^−/−^ mice on a C57BL/6 background [66] were originally purchased from Jackson laboratory (Bar Harbor, ME) and then bred in-house for experimental use. Wild type C57BL/6 mice were purchased from the National cancer institute (NCI, Frederick, MD). RAG-1^−/−^ IL-1R^−/−^ were generated by crossing C57BL/6 RAG-1^−/−^ to C57BL/6 IL-1R^−/−^ mice [67] purchased from Jackson laboratory (Bar Harbor, ME). The RAG-1^−/−^ IL-1R^−/−^ genotype was confirmed via PCR. All mice used in experiments were age matched.

### Parasite infections

Mice were infected percutaneously via tail exposure to water containing 160 *S. mansoni* cercariae (Puerto Rican strain) shed from infected *Biomphalaria glabrata* snails. Infections were terminated at 6 weeks post infection (p.i.). Worms were perfused from the portal system and immediately fixed in 4% neutral buffered formaldehyde. Male and female worms were counted and photographed at 20×magnification using a Nikon D80 10.0 megapixel digital camera attached to a Zeiss trinocular dissecting microscope. Worm growth was assessed by measuring the length of male worms from digital micrographs using Image J software (http://rsb.info.nih.gov/ij), as described previously [4]. Only male worms were measured as female growth is dependent upon receiving developmental cues from pairing with maturing males [68]. Length of male worms was compared to that of worms recovered from wild type C57BL/6 mice at 6 weeks p.i., as reported in previous publications [1], [4], [69] and in unpublished data. Fecundity of the parasites was assessed by calculating egg production per worm pair from liver egg burdens, as described previously [4].

### Measurement of liver inflammation and coagulative necrosis

C57BL/6 wild type and RAG-1^−/−^ mice were infected with cercariae as described above. At 4 weeks p.i., mice were sacrificed and their livers removed and immediately fixed in 35 ml of 4% neutral buffered formaldehyde. Liver sections were cut and stained with hematoxylin and eosin stain (H&E stain, Histoserv INC.,Germantown, MD). Slides were digitally scanned using the Hamamatsu Nanozoomer 2.0RS (Hamamatsu City, Japan). Tissue sections were analyzed using the Nanozoomer digital pathology (NDP) software. At low magnification, areas of liver tissue measuring 20 mm^2^ total area were randomly selected. The selected areas were then scanned at 5×magnification and the area occupied by inflammatory infiltrate was measured. For each liver, sections were obtained at 3 different levels and measurements were taken for at least 3 different tissue sections. The percentage area of inflammation was determined by summing up the area occupied by inflammatory infiltrate and dividing it by the total area examined. The percentage area occupied by coagulative necrosis was determined by the same method.

### Acetaminophen (AAP) and D-(+)-galactosamine hydrochloride (GalN) treatment

RAG-1^−/−^ mice were infected with cercariae as described above. Mice received weekly intraperitoneal (i.p.) injections of AAP (Sigma-Aldrich, St Louis, MO) at a dose of 5 mg/mouse dissolved in 100 µl 30% DMSO for the first 3 weeks. Mice then received AAP at a dose of 10 mg/mouse dissolved in 30% DMSO for the remaining 3 weeks. Control mice received weekly i.p. injections of 30% DMSO. D-GalN (MP Biomedicals, Solon, OH) -treated mice received biweekly i.p. injections at a dose of 10 mg/mouse for 6 weeks, using PBS without calcium and magnesium as a vehicle. Control mice received biweekly i.p. injections of PBS (Mediatech, Manassas, VA) alone. At 6 weeks p.i. mice were euthanized, H&E staining of livers was performed to confirm liver inflammation in treated mice, and parasite parameters were determined as described above.

### Monosodium urate (MSU), alum, LPS and poly I:C treatment

RAG-1^−/−^ mice were infected with cercariae as described above. Mice received biweekly i.p. injections of MSU (Invivogen, San Diego, CA) at a dose of 500 µg/mouse, Imject Alum (Thermo Scientific, Rockford, IL) at a dose of 1 mg/mouse, ultrapure LPS, *E.coli* 0111:B4 (Invivogen) at a dose of 20 µg/mouse, or poly I:C 20 µg or 40 µg/mouse. Control mice received biweekly i.p. injections of PBS without calcium and magnesium. At 6 weeks p.i., mice were euthanized and parasite parameters were determined as described above.

### Anti-TNF-α treatment

RAG-1^−/−^ mice were infected with cercariae as described above. Mice received weekly i.p. injections of Adalimumab (Abbott, Chicago, IL) at a dose of 100 µg/mouse, using PBS without calcium and magnesium as a vehicle. Control mice received weekly i.p. injections of PBS alone. At 6 weeks p.i., mice were euthanized and parasite parameters were determined as described above.

### IL-4 complex treatment

RAG-1^−/−^ mice were infected with cercariae as described above. Mice received weekly i.p. injections of 5 µg IL-4 (Peprotech, Rocky Hill, New Jersey) complexed to 25 µg anti-IL-4 antibody 11B11 (BioXCell, West New Lebanon, New Hampshire) [70]. Control mice received weekly i.p. injections of the isotype control antibody HRPN (BioXCell). At 6 weeks p.i., mice were euthanized, livers were removed for histology, and parasite parameters were determined as described above.

### RNA isolation, purification, and real-time PCR

RNA was isolated from the spleens and/or livers of wild type or RAG-1^−/−^ mice. After removal, tissues were immediately placed in 1 ml RNA-BEE (Tel-Test, Friendswood, Texas), homogenized, snap-frozen in liquid nitrogen and stored at −80°C until isolation of total RNA, following manufacturer's instructions. RNA was further purified following the RNeasy mini protocol for RNA cleanup (Qiagen, Valencia, California). Purified RNA was quantified using a ND-1000 spectrophotometer (Nanodrop, Wilmington, DE). 2 µg of RNA was used for cDNA preparation using a high capacity RNA to cDNA kit (Invitrogen, Grand Island, New York) following manufacturer's instructions. Real time PCR was performed with a MJ Research Chromo4 PTC-200 thermocycler unit (Bio-Rad, Hercules, CA) using Taqman gene expression assays and TaqMan gene expression master mix (Invitrogen) following manufacturer's instructions. Assays for the following mRNAs were performed: rsp29, GAPDH, IL-1β, TNF-α, CCL2, Relm-α, and YM1. Expression of genes of interest was normalized to the expression of GAPDH or rsp29 and fold changes in expression were calculated following the 2^−ΔΔCT^ method [71].

### Statistical analysis

All statistical analyses were performed using GraphPad Prism Inc. version 4.0 software (San Diego, California). Significant differences between two groups were determined using a student's unpaired T-test with Welch's correction or a Mann-Whitney test. Significant differences between 3 or more groups were determined using a Kruskal-Wallis test followed by a Dunn's multiple comparison test. *P* values of less than 0.05 were considered significant. 3–5 mice were used per experimental group and all experiments were performed at least twice.

## Supporting Information

Figure S1 **Acetaminophen (AAP) and D-galactosamine (GalN) induce liver necrosis and inflammation in RAG^−/−^ mice.** Representative H&E-stained liver sections from RAG^−/−^ mice treated throughout pre-patent infection with acetaminophen (A) or D-galactosamine (B). Arrowheads indicate areas of necrosis. Arrows indicate infiltrating inflammatory cells. Scale bars in A and B are 300 µm in length.
(EPS)

Figure S2 **Regulation of pro-inflammatory cytokine transcription requires repeated administration of MSU.** Groups of *S. mansoni*-infected RAG^−/−^ mice were either repeatedly treated with MSU throughout pre-patent infection, until 6 weeks p.i., and were euthanized 18 hours after the last dose of MSU (A–C), as described in Materials and Methods, or received a single 500 µg dose of MSU 18 hours prior to euthanasia (D–F). At euthanasia, livers were removed for analysis of IL-1α (A, D), IL-1β (B, E) and CCL2 (C, F) mRNA content by real-time PCR. Horizontal bars represent mean values for each experimental group. *P* values were calculated using the Mann Whitney test.
(EPS)
